# Chinese college students COVID-19 phobia and negative moods: Moderating effects of physical exercise behavior

**DOI:** 10.3389/fpubh.2022.1046326

**Published:** 2022-12-01

**Authors:** Shan-shan Han, Ya-hui Han, Wen-xia Tong, Guang-xu Wang, You-zhi Ke, Shu-qiao Meng, Qiang Guo, Zhong-lei Cui, Jun-yong Zhang, Yu-peng Ye, Yao Zhang, Ya-xing Li, Bo Li

**Affiliations:** ^1^Institute of Sports Science, Nantong University, Nantong, China; ^2^Institute of Sports Science, Kyunggi University, Suwon, South Korea; ^3^Physical Education College, Yangzhou University, Yangzhou, China; ^4^College of Physical Education, Henan Normal University, Xinxiang, China; ^5^School of Physical Education, Shanghai University of Sport, Shanghai, China; ^6^Physical Education College, Shangqiu University, Shangqiu, China; ^7^Physical Education College of Shangqiu Normal University, Shangqiu, China; ^8^School of Physical Education, Henan University of Economics and Law, Zhengzhou, China; ^9^School of Physical Education, Jinggangshan University, Ji'an, China; ^10^Institute of Sports and Health, Zhengzhou Shengda University, Zhengzhou, China

**Keywords:** specific phobias, negative moods, moderating effects, COVID-19, mental health, college students

## Abstract

**Objective:**

We investigated the effects of COVID-19 fear on negative moods among college students, and assessed the efficacy of physical exercise behavior as a moderator variable.

**Methods:**

This was a cross-sectional study. Students from three colleges and universities in Shangqiu City, Henan Province and Yangzhou City, Jiangsu Province were enrolled in this study, which was performed during the COVID-19 pandemic using an online questionnaire. A total of 3,133 college students completed the questionnaire. Measurement tools included the COVID-19 Phobia Scale (C19P-S), Depression-Anxiety-Stress Self-Rating Scale (DASS), and the Physical Activity Behavior Scale (PARS-3).

**Results:**

During the COVID-19 pandemic, the rates of depression, anxiety, and stressful negative moods among college students were 35.5, 65.5, and 10.95%, respectively; there was a positive correlation between COVID-19 fear and negative moods among college students (*r* = 0.479, *p* < 0.001), which was negatively correlated with physical exercise behavior (*r* = −0.4, *p* < 0.001); the regulating effects of physical exercise behavior were significant (ΔR2 = 0.04, *p* < 0.001).

**Conclusion:**

The rate of negative moods among college students is high, and the fear for COVID-19 is one of the key factors that lead to negative moods. Physical exercise can modulate the impact of COVID-19 fear among college students on negative moods. Studies should elucidate on mental health issues among different populations during the COVID-19 pandemic.

## Introduction

Coronavirus disease 2019 (COVID-19) is a global pandemic (1) whose rapid spread has led to an increase in negative news. Knowledge on health among people in China is low, which hampers efforts that are aimed at establishing the onset of epidemics to design appropriate control strategies (2). Therefore, COVID-19 is a major infectious disease that is associated with various stressful psychological disorders, such as worry, anxiety, depression, helplessness, panic, and anger among others. Its pathological symptoms include tachycardia, body tremors, anorexia, and difficulty falling asleep. Studies were aimed at developing suitable approaches to reduce psychological stress levels during global pandemics, such as COVID-19, and to improve immunity (3).

College students are in the transition stage from adolescence to adulthood. Due to various external factors (such as interpersonal relationships, study pressures, campus life, etc.) and their own bad cognition, these students have frequently experienced psychological problems with extreme cases such as suicide and self-harm (4, 5). Among them, incidences of emotional behavior problems due to negative moods are higher and are more harmful (6, 7). Negative moods are correlated with perception of various kinds of pain and unpleasantness, and they often lead to intense behaviors (such as suicidal behaviors) and physiological reactions (such as crying) (8, 9). Among the connotative indicators of negative moods, depression, anxiety and stress are the most common among college students (10). Depression, anxiety, and stress are interconnected and often co-occur, leading to extreme behavioral problems such as suicide among college students (11, 12). Therefore, studies on related factors that induce negative moods are important to improve the mental health of college students.

Epidemics such as the H1N1 virus and the SARS-coronavirus had severe negative psychological effects, and caused phobia in people (13–15). Phobias are defined by persistent and excess fear of objects, situations, and phenomena. According to the American Psychiatric Association (APA) Diagnostic and Statistical Manual of Mental Disorders (DSM-V) criteria, phobias include social phobia, agora phobia and specific phobia (16). The phobia caused by COVID-19 is a specific phobia (17–19). Individual temperament, genetic and physiological factors, as well as environmental conditions are predisposing factors for specific phobias (16). Major infectious disease outbreaks, such as the COVID-19 pandemic, are among environmental triggers of phobias. Globally, specific phobias are the most common psychiatric disorders (20, 21). Persistent specific phobias can trigger suicidal tendencies, major depressive disorders and anxiety disorders among others (22, 23). Therefore, it is necessary to pay attention to phobias during major public health emergencies.

Physical exercise is effective for coping with psychological stress. Different exercise programs, exercise durations and exercise intensity have different effects on the body's response to psychological stress. Moderate-intensity physical exercise enhances immune functions, which enhances adaptability to chronic and acute psychological stress (24–27). Studies on empirical interventions of mental health challenges among college students through sports programs verified the above conclusions (28, 29). Therefore, this study introduced physical exercise behavior as a moderator variable.

**Figure 1:**
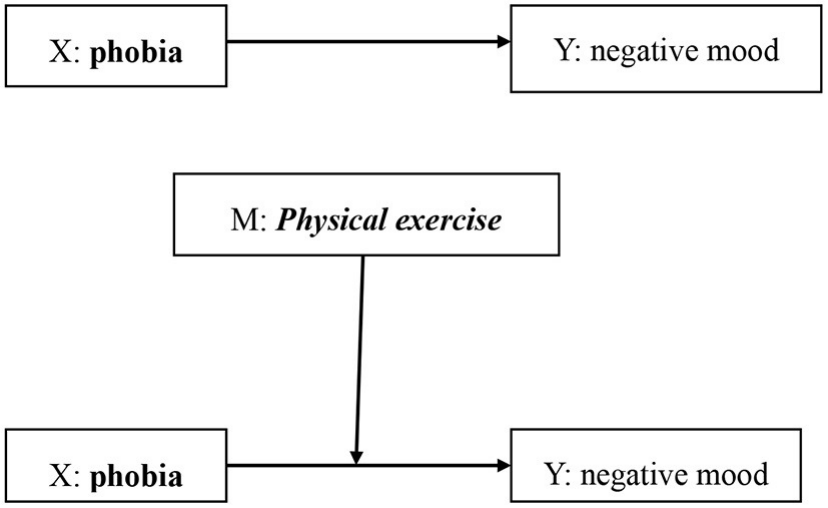
Conceptual model of this study.

Based on literature review, college students were used as study participants, “COVID-19 fear and negative moods” was the breakthrough point, and physical exercise behavior was introduced as a moderator variable. The hypotheses in this study were: H1: There is a significant positive correlation between COVID-19 fear and negative moods among college students; H2: Physical exercise behavior can moderate the impact of COVID-19 fear on negative moods among college students (Figure 1). The purpose of this study is to verify the above two null hypotheses. To test whether physical exercise behavior of college students can alleviate negative emotional problems caused by COVID-19 fear. This study is of great significance as it elucidates on inducing factors of negative moods such as anxiety, depression and stress to promote the mental health of college students.

## Materials and methods

### Participants

We used an online questionnaire, which is published online through the “Questionnaire Star” software. Eight questionnaire investigators were trained in this study, and the identity of every investigator was a “student counselor”. Students from three universities; Yangzhou University in Yangzhou City, Jiangsu Province, Shangqiu College in Shangqiu City, Henan Province, and Shangqiu Normal University were enrolled in the questionnaire survey [Yangzhou City, Jiangsu Province (investigation time: October 2021) and Shangqiu City, Henan Province (investigation time: May 2021)]. The universities under investigation have epidemic prevention and control plans to restrict students' out-of-school activities. Questionnaires were distributed and collected through the online platform (Questionnaire Star).

The main questionnaire distribution procedures were: (i) Unified release of standardized filling instructions, questionnaires and reward mechanism descriptions; (ii) Subjects filled in anonymously, of which 151 people filled in their real names, and those who anonymously completed their questionnaire get paid after completing the questionnaire. The IP address was set to allow filling only once. This part of the sample was set as sample 1, and the questionnaire filling time was 5 working days. (iii) After an interval of 10 working days, real names were filled in to complete the second filling. This sample was set as sample 2, and the questionnaire filling time was 5 working days. Sample 2 was only used for test-retest reliability assessment of the scale.

### Measurements

#### COVID-19 phobia scale

The COVID-19 phobia scale (C19P-S) is a specific phobia scale specifically developed to assess the fear of COVID-19. Arpaci et al. (17), at the recommendation of the APA, developed a test item based on the current C19P-S that corresponds to the DSM-V's specific phobia diagnostic criteria. The C19P-S has a total of 20 items, including 4 factors of psychology, physiology, economy and society, each of which can be individually applied. Since the scale has no precedent for its application in the Chinese population, we conducted translation and adaptation tests on the scale.

Chinese translation of C19P-S was performed *via* the “round-trip translation method”. First, a graduate student majoring in English and a graduate student majoring in psychology independently translated the questionnaire, after which the two graduate students discussed and obtained the first draft of the translation. Then, the first draft was handed over to a Ph.D. in psychology (associate professor, doctoral supervisor) for English back-translation. Finally, a Chinese scholar who is familiar with Chinese and English and who lives in an English-speaking country was invited to review the content of the back-translation, conduct Chinese translation, and finally determine the Chinese final draft of C19P-S. The C19P-S uses a Likert 5-point scale ranging from 1 (strongly disagree) to 5 (strongly agree). The higher the score, the higher the fear of COVID-19.

Based on classical measurement theory, we conducted a reliability and validity test for C19P-S. Reliability and validity tests showed that the means for all items in C19P-S were between 1.79 and 2.53, while their standard deviations were between 0.97 and 1.39. Correlations r between each item score and total score were above 0.78 (Table 1), indicating that all items have a good discrimination degree. Exploratory factor analysis showed that: KMO = 0.97, Bartlett sphericity test was significant (χ2 = 64,554.62, df = 190, *p* < 0.001), indicating that factor analysis was suitable. Exploratory factor analysis was performed by principal component analysis. Based on characteristic root >1, 4 factors were extracted. Combined with the gravel diagram, the fifth eigenvalue was established to be an inflection point, after which the trend became flat, and was considered appropriate for extraction of the 4 factors. Therefore, exploratory factor analysis was performed after setting the number of factors to 4. Factor loading for each item was between 0.42 and 0.89 (Table 2), which indicated that the scale had good construct validity. The test-retest reliability coefficient was between 0.69 and 0.95, indicating that the scale had good test-retest reliability. Cronbach's alpha coefficients for the four sub-test scales were 0.79, 0.81, 0.85, and 0.78, respectively, while the overall Cronbach's alpha coefficient for the scale was 0.69, which indicated that the scale had good internal consistency. Overall, based on the classical measurement theory, it was determined that the C19P-S could be used in the Chinese college student population.

**Table 1:** Summary of C19P-S descriptive analysis, correlation analysis, and exploratory factor analysis results.

**Dimension**	**Item**	**Descriptive analysis**		**Related analysis**	**Exploratory factor analysis**				
		***M***	**SD**	***r***	**Factor 1**	**Factor 2**	**Factor 3**	**Factor 4**	**Commonality**
**Psychology**									
	PS_1	2.181	1.178	0.831^**^		0.741			0.760
	PS_2	2.532	1.394	0.804^**^		0.811			0.781
	PS_3	2.338	1.269	0.849^**^		0.842			0.884
	PS_4	2.325	1.258	0.859^**^		0.823			0.862
	PS_5	2.413	1.276	0.852^**^		0.820			0.856
	PS_6	2.375	1.274	0.823^**^		0.738			0.755
**Physiological**									
	PH_1	1.845	1.008	0.804^**^	0.861				0.889
	PH_2	1.806	0.981	0.788^**^	0.883				0.924
	PH_3	1.795	0.968	0.789^**^	0.889				0.933
	PH_4	1.830	0.995	0.811^**^	0.868				0.938
	PH_5	1.857	1.030	0.822^**^	0.842				0.916
**Economy**									
	EC_1	1.938	1.065	0.854^**^				0.451	0.874
	EC_2	1.989	1.071	0.873^**^				0.611	0.893
	EC_3	2.018	1.077	0.874^**^				0.623	0.876
	EC_4	1.991	1.052	0.870^**^				0.588	0.878
**Society**									
	SO_1	2.172	1.181	0.881^**^			0.420		0.859
	SO_2	2.442	1.293	0.809^**^			0.812		0.896
	SO_3	2.517	1.303	0.776^**^			0.813		0.882
	SO_4	2.203	1.172	0.867^**^			0.564		0.800
	SO_5	2.194	1.153	0.881^**^			0.538		0.820

** *P* < 0.001.

**Table 2:** Distribution of research objects (sample 1).

**Index**	**Number**	**Percentage (%)**
**Sex**		
Male	1,311	41.8
Female	1,822	58.2
**Area**		
Shang Qiu	1,979	63.2
Yang Zhou	1,154	36.8
**Grade**		
First	1,492	47.6
Second	1,086	34.7
Third	555	17.7
Overall	3,133	100.0

#### Depression, anxiety, and stress scale

The depression, anxiety and stress scale (DASS) is based on the three-dimensional model proposed by Clark and Watson, which argues that depression and anxiety have both unique and common symptoms (30). In this study, the 21-Item Depression Anxiety and Stress Scale (DASS-C21) was adapted by Wen, specifically for Chinese college students (31). The scale uses the Likert 4-point scoring standard, with corresponding scores ranging from 0 to 3, and higher scores indicating higher levels of negative moods.

The reliability and validity of DASS-C21 among Chinese college students is high (31). The norm for DASS-C21 college students is: depression score ≤9 is normal, 10–13 is mild, 14–20 is moderate, 21–27 is severe, ≥28 is very severe. Anxiety score ≤ 7 is considered normal, 8–9 is mild, 10–14 is moderate, 15–19 is severe, and ≥20 is very severe. A stress score of ≤14 is considered normal, 15–18 is considered mild, 19–25 is considered moderate, 26–33 is considered severe, and ≥34 is considered very severe (31). DASS-C21 is often used to assess negative mood symptoms in clinical practice.

The reliability and validity of DASS-C21 among Chinese college students are relatively high (31). The overall Cronbach's alpha coefficient and test-retest reliability of the scale are 0.912 and 0.751, respectively, while the average correlation coefficient between items is 0.338. The Pearson correlation coefficient of the total score of the scale is between 0.895 and 0.910, while the correlation coefficient between the scores for each subscale is 0.708–0.741 (*p* < 0.01). CFI = 0.914, IFI = 0.909, TLI = 0.894, RMSEA = 0.059 (31). In general, DASS-C21 is suitable for Chinese college students.

#### Physical activity rating scale

The physical activity rating scale (PARS-3) was compiled by the Japanese scholar, Takao Hashimoto, and was subsequently completed by Liang in China. Physical exercise volume was assessed with respect to exercise intensity, frequency, and time of one exercise activity to measure physical exercise participation (32).

**Physical exercise volume score = intensity × (time – 1) × frequency (2)**

Each parameter was evaluated using five score levels. Level standards were: small exercise volume ≤19 points, moderate exercise volume = 20–42 points, and large exercise volume ≥43 points (32). Re-test reliability of this scale was 0.820. Follow-up related research showed that internal consistency reliability of PARS-3 was Cronbach's α = 0.85. The value of the scale was used to reflect physical exercise behaviors of college students.

### Statistical analysis

We used SPSS 25 for analysis. The main calculation steps for C19P-S reliability and validity were: (i) Determination of the mean and standard deviations for each item, and using the Spearman rank correlation to assess correlations between the score for each item and total score, so as to perform item analysis; (ii) Using exploratory factor analysis to test the construct validity of the scale; (iii) Performing the Spearman rank correlation analysis to assess the test-retest reliability; (iv) Using the Cronbach α coefficient to test the internal consistency reliability of the scale.

In this study, descriptive data were used to analyze the current situation of college students' COVID-19 fear, negative moods and physical exercise behaviors, with one-way ANOVA performed to analyze differences in COVID-19 fear among students of different gender and grades. The effect size was η^2^; the chi-square test was performed to assess differences in negative moods and physical exercise behaviors of students of different genders and grades (effect size is based on Cramer's V coefficient). The Pearson simple correlation analysis was used to assess correlations among COVID-19 fear (including four sub-tests of physiology, psychology, economy and society), negative moods (including three sub-tests of depression, anxiety and stress) and physical exercise. Linear regression analysis was performed to verify the moderating effects of physical exercise behaviors. The three variables were standardized (Z-score) before the assessments of moderating effects.

The procedure for testing the moderating effect is as follows. (i) Negative emotion is the dependent variable, and all control variables are added to form M0. (ii) On the basis of M0, the independent variable COVID-19 phobia and the moderating variable physical exercise behavior were added to form M1. (iii) On the basis of M1, the interaction term (independent variable ^*^ moderating variable) is added to form M2. In the interpretation of the results, we mainly refer to the following indicators. The F-value is used to determine whether the model is meaningful, and when *p* < 0.05, it indicates that the model is meaningful. R^2^ represents how well the model fits, and the closer to 1, the better. ΔR^2^ stands for model change and is used to explain the explanatory force of the model. In the step-by-step interpretation of the above statistical indicators, when all the tests are passed, ΔR^2^ can be used to verify the adjustment effect of the interaction between the independent variable and the adjustment variable, and the larger the value of ΔR^2^, the more significant the adjustment effect (33).

## Results

### Descriptive analysis

Table 3 shows that there were significant gender differences with regards to psychological, physiological, economic, social and total scores of COVID-19 fear among college students. Specifically, the score for women was significantly higher than that of men (*p* < 0.05), implying that women are more afraid of COVID-19. Differences in psychological scores of college students in different grades were insignificant (*p* = 0.192), with the highest score in the first grade and the lowest score in the second grade. There were significant differences in scores among different grades of physiology, economy, society and total scores (*p* < 0.05). The highest score was in the second grade while the lowest score was in the first grade, in contrast to the psychological score and trend.

**Table 3:** COVID-19 fear among students of different gender and grade.

	**Overall**		**Sex**				**Grade**					
			**Male (*****n*** = **1,311)**		**Female (*****n*** = **1,822)**		**First (*****n*** = **1,492)**		**Second (*****n*** = **1,086)**		**Third (*****n*** = **555)**	
	**M**	**SD**	**M**	**SD**	**M**	**SD**	**M**	**SD**	**M**	**SD**	**M**	**SD**
**Psychology**												
Original score	14.030	6.456	12.676	6.382	15.004	6.335	13.796	6.278	14.269	6.651	14.248	6.550
Standard score	2.339	1.076	2.113	1.064	2.501	1.056	2.299	1.046	2.378	1.108	2.375	1.092
*F*			98.782				1.649					
*p*			<0.001				0.192					
η^2^			0.031				0.001					
**Physiological**												
Original score	8.700	4.214	8.507	4.449	8.843	4.034	8.338	3.886	9.127	4.501	8.891	4.434
Standard score	1.741	0.843	1.701	0.890	1.769	0.807	1.668	0.777	1.825	0.900	1.778	0.887
*F*			3.993				12.184					
*p*			0.045				<0.001					
η^2^			0.001				0.008					
**Economy**												
Original score	7.610	3.701	7.132	3.690	7.958	3.672	7.336	3.498	7.925	3.840	7.783	3.928
Standard score	1.904	0.925	1.783	0.923	1.990	0.918	1.834	0.874	1.981	0.960	1.946	0.982
*F*			36.154				8.05					
*p*			<0.001				<0.001					
η^2^			0.011				0.005					
**Society**												
Original score	11.030	5.014	10.092	4.999	11.699	4.918	10.706	4.795	11.337	5.142	11.343	5.318
Standard score	2.206	1.003	2.018	1.000	2.340	0.984	2.141	0.959	2.267	1.028	2.269	1.064
*F*			77.203				5.453					
*p*			<0.001				0.004					
η^2^				0.024				0.003				
**Overall**												
Original score	41.381	17.356	38.490	17.832	43.461	16.704	40.209	16.306	42.658	18.260	42.031	18.087
*F*			68.803				5.729					
*p*			<0.001				<0.001					
η^2^			0.020				0.004					

In Table 4, the normal evaluation of depression in the negative moods of college students included in the analysis was 64.48% of the total sample, about 35.5% of college students had depressive symptoms, and about 13.25% of the total sample was moderate or above. College students in the anxiety test accounted for 34.50% of the total sample. About 65.50% of college students had symptoms of anxiety. Incidences of severe and very severe anxiety were 5.68 and 1.79%, respectively. The stress test showed that 89.05% of college students were normal, only 10.95% had severe stress, 3% had moderate or above stress, and there were no college students with very severe stress. Physical exercise assessment revealed that 66.14% of college students performed small amounts of exercise, 16.01% performed moderate amounts of exercise, while 17.81% of total sample were engaged in large amounts of exercise.

**Table 4:** Status quo and gender and grade differences in NM and physical exercise behaviors among college students.

**Index**	**Overall**		**Sex**				**Grade**					
			**Male (*****n*** = **1,311)**		**Female (*****n*** = **1,822)**		**First (*****n*** = **1,492)**		**Second (*****n*** = **1,086)**		**Third (*****n*** = **555)**	
	***n***	**%**	***n***	**%**	***n***	**%**	***n***	**%**	***n***	**%**	***n***	**%**
**Depression**												
Normal	2,020	64.475	871	66.438	1,149	63.063	1,010	67.694	644	59.300	366	65.946
Mild	698	22.279	249	18.993	449	24.643	336	22.520	245	22.560	117	21.081
Moderate	361	11.523	159	12.128	202	11.087	135	9.048	161	14.825	65	11.712
Severe	43	1.372	23	1.754	20	1.098	11	0.737	28	2.578	4	0.721
Very serious	11	0.351	9	0.686	2	0.110	0	0.000	8	0.737	3	0.541
χ2			22.608				53.776					
*p*			<0.001				<0.001					
Cramer's V			0.085				0.093					
**Anxiety**												
Normal	1,081	34.504	522	39.817	559	30.681	506	33.914	369	33.978	206	37.117
Mild	858	27.386	330	25.172	528	28.979	441	29.558	267	24.586	150	27.027
Moderate	960	30.642	353	26.926	607	33.315	446	29.893	350	32.228	164	29.550
Severe	178	5.681	74	5.645	104	5.708	87	5.831	62	5.709	29	5.225
Very serious	56	1.787	32	2.441	24	1.317	12	0.804	38	3.499	6	1.081
χ2			38.028				36.104					
*p*			<0.001				<0.001					
Cramer's V			0.110				0.076					
**Stress**												
Normal	2,790	89.052	1,162	88.635	1,628	89.352	1,363	91.354	931	85.727	496	89.369
Mild	249	7.948	95	7.246	154	8.452	104	6.971	105	9.669	40	7.207
Moderate	83	2.649	44	3.356	39	2.141	25	1.676	42	3.867	16	2.883
Severe	11	0.351	10	0.763	1	0.055	0	0.000	8	0.737	3	0.541
Very serious	0	0.000	0	0.000	0	0.000	0	0.000	0	0.000	0	0.000
χ2			16.574				30.404					
*p*			0.001				<0.001					
Cramer's V			0.073				0.070					
**Physical exercise**												
Small	2,072	66.135	608	46.377	1,464	80.351	1,119	75.000	626	57.643	327	58.919
Moderate	503	16.055	286	21.815	217	11.910	210	14.075	194	17.864	99	17.838
Large	558	17.810	417	31.808	141	7.739	163	10.925	266	24.494	129	23.243
χ2			427.650				117.044					
*p*			<0.001				<0.001					
Cramer's V			0.369				0.137					

The rates of depression and anxiety were significantly higher among women than in men (Cramer's V = 0.085, *p* < 0.001). The rate of anxiety was significantly higher in males than in females (Cramer's V = 0.11, *p* < 0.001). Men and women had comparable stress rates. Overall, the negative moods among college students included in the analysis were serious, and were more severe among women than in men. There were significant differences in physical exercise behaviors among college students of different sex (Cramer's V = 0.369, *p* < 0.001). Proportions of moderate and heavy exercises among male students were higher than in female students. Differences in rates of negative moods and physical exercise behaviors among college students of different grades were also significant (*p* < 0.001 for both). The proportion of students in the second grade who exercised moderately and heavily was greater than that of students in the first and third grades.

### Correlation analysis

Table 5 shows that the correlation coefficient between total score of C19P-S scale and physical exercise behaviors for college students was −0.1400 (*p* < 0.001). The four subscales of C19P-S were negatively correlated with physical exercise behaviors of college students. Specifically, the correlation coefficient between college students' physiological phobia and physical exercise behavior was −0.577 (*p* < 0.001), that between college students' economic phobia and physical exercise behavior was −0.393 (*p* < 0.001), while the correlation coefficient between college students' social phobia and physical exercise behavior was −0.393 (*p* < 0.001). The correlation coefficient for behavior was −0.485 (*p* < 0.001). From the perspective of negative moods, the correlation coefficient between DASS total score and physical exercise behavior was −0.483 (*p* < 0.001), that between depression and physical exercise behavior in the subscale was −0.482 (*p* < 0.001), anxiety and physical exercise correlation coefficient of behavior was −0.577 (*p* < .001). The correlation coefficient between stress and physical exercise behavior was −0.480 (*p* < 0.001). The correlation coefficient between C19P-S and DASS total scores was 0.479 (*p* < 0.001). Moreover, there were significant correlations between the four dimensions of C19P-S and the three dimensions of negative mood (*r* = 0.372–0.443, *p* < 0.001). The above results suggest that further moderating effect analysis can be conducted on COVID-19 phobia, negative moods and physical exercise behaviors among college students.

**Table 5:** Correlations between COVID-19 fear, NM, and physical exercise behaviors among college students.

		**Physiological**	**Economy**	**Society**	**C19P_S**	**Depression**	**Anxiety**	**Stress**	**DASS**
Psychology	*r*	0.624^**^	0.709^**^	0.759^**^	0.894^**^	0.374^**^	0.413^**^	0.421^**^	0.420^**^
	*p*	<0.001	<0.001	<0.001	<0.001	<0.001	<0.001	<0.001	<0.001
Physiological	*r*	1	0.854^**^	0.689^**^	0.856^**^	0.429^**^	0.443^**^	0.412^**^	0.446^**^
	*p*		<0.001	<0.001	<0.001	<0.001	<0.001	<0.001	<0.001
Economy	*r*	0.854^**^	1	0.813^**^	0.919^**^	0.427^**^	0.440^**^	0.434^**^	0.452^**^
	*p*	<0.001		<0.001	<0.001	<0.001	<0.001	<0.001	<0.001
Society	*r*	0.689^**^	0.813^**^	1	0.912^**^	0.372^**^	0.396^**^	0.403^**^	0.407^**^
	*p*	<0.001	<0.001		<0.001	<0.001	<0.001	<0.001	<0.001
C19P_S	*r*	0.856^**^	0.919^**^	0.912^**^	1	0.442^**^	0.470^**^	0.465^**^	0.479^**^
	*p*	<0.001	<0.001	<0.001		<0.001	<0.001	<0.001	<0.001
Depression	*r*	0.429^**^	0.427^**^	0.372^**^	0.442^**^	1	0.868^**^	0.875^**^	0.953^**^
	*p*	<0.001	<0.001	<0.001	<0.001		<0.001	<0.001	<0.001
Anxiety	*r*	0.443^**^	0.440^**^	0.396^**^	0.470^**^	0.868^**^	1	0.894^**^	0.959^**^
	*p*	<0.001	<0.001	<0.001	<0.001	<0.001		<0.001	<0.001
Stress	*r*	0.412^**^	0.434^**^	0.403^**^	0.465^**^	0.875^**^	0.894^**^	1	0.965^**^
	*p*	<0.001	<0.001	<0.001	<0.001	<0.001	<0.001		<0.001
DASS	*r*	0.446^**^	0.452^**^	0.407^**^	0.479^**^	0.953^**^	0.959^**^	0.965^**^	1
	*p*	<0.001	<0.001	<0.001	<0.001	<0.001	<0.001	<0.001	
PARS-3	*r*	−0.377^**^	−0.393^**^	−0.485^**^	−0.400^**^	−0.482^**^	−0.577^**^	−0.480^**^	−0.483^**^
	*p*	<0.001	<0.001	<0.001	<0.001	<0.001	<0.001	<0.001	<0.001

** *P* < 0.001.

### Moderating effects test

Table 6 shows the moderating effects of physical exercise behavior. Table 6 shows that in model 1, the explanation rate of independent variables and moderator variables for the dependent variable is 23%, and in model 2, the predictive power increases by 4%. These findings imply that under the condition that independent and moderator variables remain unchanged, an increase of 4% is the predictive ability of the interaction term for the dependent variable, that is, the contribution rate of the moderating effect. The significant F change was <0.001 in model 1 and 0.034 (<0.05) in model 2, indicating that the independent and moderator variables were significant in predicting the dependent variable. Further, *p* values in ANOVA were all < 0.001, indicating that the moderating effect of the moderator variable on the independent variable was significant.

**Table 6:** Analysis of the moderating effects of physical exercise behaviors.

**Model**	**Model summary**					**ANOVA**	
	**R^2^**	**ΔR^2^**	**df 1**	**df 2**	**Sig. change**	***F***	***p***
1	0.230	0.230	2	3,130	<0.001	468.422	<0.001
2	0.230	0.040	1	3,129	0.034	312.232	<0.001

## Discussion

In this study, we established that college students mainly indulge in small amounts of exercise (about 66.14% of the students in this study), and exercise level among males was significantly higher than among females. These findings are in tandem with those of previous studies. After the COVID-19 pandemic, the proportion of college students engaging in small exercises increased significantly, which had a significant negative impact on college students' physical exercise. A previous study reviewed the impact of the outbreak of the new crown epidemic on physical activities among college students. Walking, intensities and total physical activity levels among college students in different countries were found to be significantly decreased (34). The COVID-19 pandemic has exacerbated the decline in physical activities of various groups of people, and the relevant epidemic prevention policies in China restricted students' outdoor physical exercises to a certain extent.

Regarding negative moods among college students, we established that the rates of depression, anxiety and stress were 35.5, 65.5, and 10.95%, respectively. Xiao et al. reported that the rate of depressive symptoms in Chinese college students during the COVID-19 pandemic was 59.35%, while that of anxiety symptoms was 54.34% (35). In their study, Xiang et al. found that during the COVID-19 pandemic, incidences of depressive and anxiety symptoms in China were 41.8 and 31.0%, respectively (36). These results differ from ours, but they all show that the rates of depression and anxiety among Chinese college students during the COVID-19 pandemic was higher.

In this study, gender had a significant impact on college students' COVID-19 phobia, physical exercise behaviors and occurrence of negative moods. In accordance with our results, gender has been significantly associated with the mental health of college students during the COVID-19 epidemic (37). Social isolation as well as the qualities of social relationships and information received were significantly associated with mental health among college students during the COVID-19 epidemic (37).

The optimal intensity of exercise is to choose the content and intensity of exercise independently based on individual differences to better stimulate exercise motivation and emotional responses, and fixed intensity exercise leads to poor emotional responses and efforts (38, 39). Regarding the form and content of exercise, Wang et al. reported that high-frequency, high-dose moderate-to-high-intensity aerobic exercise combined with strength training and various coordination training has better intervention effects on negative moods (40). Home or school isolation during the COVID-19 pandemic negatively impacted college students' mental health (41–43). Moreover, due to the closed and depressing environment, lack of venues, equipment, and training partners, the duration and intensity of most students' exercise decreased (44), which may have reduced the effectiveness of physical exercise in preventing and improving negative moods. In addition, a longer period of epidemic isolation exacerbated the worsening of negative moods among college students. The results of this study validate the positive effects of physical exercise on mental health.

College students' COVID-19 phobia was positively correlated with negative moods. Hypothetically, COVID-19 phobia affects students' cognitive thinking and emotional cognition, thereby increasing the detection rate of negative moods among college students. This study confirmed the negative correlation between COVID-19 phobia and physical exercise behaviors among college students, that is, the higher the degree of fear, the lower the level of physical exercise. All four dimensions of the C19P-S scale were negatively correlated with physical exercise behaviors. The negative correlation with physical exercise behavior is that fear contains factors that can make students feel afraid, so that students' physical exercise behavior is weakened. In addition, isolation or blockade in movement during the pandemic may have resulted in psychological, physical, social and economic fears among students, which may be the main reason for the reduction in physical exercise.

Our findings validated the null hypothesis H1; the rates of COVID-19 phobia and negative moods among college students were significantly and positively correlated. The more the rates of negative moods among college students, the less the physical exercise. College students independently face academic, employment, social, emotional and other issues, thus, they were more likely to have negative moods during the pandemic. Physical exercise is important for reducing negative moods among college students and is positively correlated with enhancement of college students' self-esteem and self-confidence (45). The negative relationship between college students' negative moods and physical exercise has been widely verified, and the time, intensity and frequency of sports are the main indicators that affect college students' negative moods (46, 47). This result provides a new perspective for combination of school sports and education to jointly improve the mental health and physical activity levels of college students. This study has certain practical significance for guiding students' psychological interventions and strengthening physical exercises in future.

The results of this study validate the null hypothesis H2; physical exercise behaviors can moderate the effects of college students' COVID-19 phobia on negative moods. The mental health of college students can be improved through physical exercise (48–50). Some studies used different sports programs to enhance the mental health of college students. Jiao et al. used Wuqinxi to enhance the mental health of female college students, they found that after a semester of interventions, depressive symptoms among students were significantly reduced (51). By changing the exercise behaviors of the subjects to increase the amounts of physical activities, such as increasing the intensity, frequency, and increasing the duration of behaviors. Negative moods decrease with increasing physical exercise levels.

Results from the moderating effects can be explained using the distraction hypothesis, which states that sustained physical activity can divert individuals from unpleasant stimuli or painful physical discomfort, thereby improving overall moods (52). The monoamine hypothesis at the physiological level states that appropriate intensities of physical activities can increase the release rate of monoamine transmitters in the human body, promote the transmission of monoamine transmitters between neurons, and accelerate the absorption of monoamine transmitters by neurons, thereby improving the moods of subjects (53). Based on these theories, studies should aim at establishing suitable approaches for promotion or improvement of mental health of college students by exercise.

The limitation of this study is that we adopted a cross-sectional study design, thus, the dose-response relationship between COVID-19 phobia and negative moods was not explored. The methodological system for assessment of causality in health research is relatively mature. For the five types of challenges in exploration of causality, such as measurement bias, omitted variables, mutual causality, common cause and selection bias, the causal relationship has been established. Inference methods include randomized controlled experiments, propensity score matching, instrumental variables, difference-in-differences, breakpoint regression designs, and individual fixed-effects models (cross-lag models, latent growth linear models, etc.) (54). Scholars can choose the methodology according to their own research expertise, and at the same time, combined with the current development of big data technology, make full use of machine learning to enhance the scientific nature of their own research, improve research efficiency, and improve the generalization and popularization of research conclusions so as to enhance academic strength for the realization of a higher level of national health.

## Conclusion

We found a high rate of negative moods among college students during the COVID-19 pandemic period, which was higher among female students compared to male students. College students' COVID-19 phobia may be one of the key factors inducing negative moods. Physical exercise can moderate the impact of COVID-19 phobia on negative moods among college students. More studies should elucidate on mental health issues in different populations during the COVID-19 pandemic.

## Data availability statement

The original contributions presented in the study are included in the article/supplementary material, further inquiries can be directed to the corresponding author.

## Ethics statement

The studies involving human participants were reviewed and approved by Nantong University. The patients/participants provided their written informed consent to participate in this study.

## Author contributions

Conceptualization: S-sH. Data curation: S-sH, BL, Y-hH, Y-zK, G-xW, S-qM, Y-xL, Z-lC, and W-xT. Formal analysis: S-sH and W-xT. Funding acquisition: S-sH, Y-zK, S-qM, Y-xL, and W-xT. Investigation: S-sH, Y-xL, and W-xT. Methodology: S-sH, G-xW, Y-xL, and W-xT. Project administration: S-sH, BL, Y-zK, G-xW, S-qM, Z-lC, and W-xT. Resources: W-xT. Software: S-sH and G-xW. Supervision: W-xT. Validation: S-qM and Z-lC. Visualization: Y-xL and W-xT. Writing—original draft: S-sH, BL, Y-hH, and Y-zK. All authors have read and agreed to the published version of the manuscript.

## Funding

This study was supported by the Jiangsu Province Education Science 14th Five-Year Plan Project (T-c/2021/108).

## Conflict of interest

The authors declare that the research was conducted in the absence of any commercial or financial relationships that could be construed as a potential conflict of interest.

## Publisher's note

All claims expressed in this article are solely those of the authors and do not necessarily represent those of their affiliated organizations, or those of the publisher, the editors and the reviewers. Any product that may be evaluated in this article, or claim that may be made by its manufacturer, is not guaranteed or endorsed by the publisher.

## References

[1] World Health Organization. Coronavirus Disease (COVID-19) Pandemic. Geneva: World Health Organization (WHO). (2021). Available online at: https://www.who.int/emergencies/diseases/novel-coronavirus-2019 (accessed December 30, 2021).

[2] Li Z Tian Y Gong Z. Health literacy and regional heterogeneities in China: a population-based study. Front Public Health. (2021) 9:603325. 10.3389/fpubh.2021.603325 34046382 PMC8144299

[3] Lou H Yan J. Psychoneuroimmune pathways and countermeasures of physical exercise in major infectious disease epidemics. China Sports Technol. (2020) 56:35–40+89. 10.16470/j.csst.2020041

[4] Xiang YT. Prevalence of depression and its relationship with quality of life among university students in Macau, Hong Kong and mainland China. Sci Rep. (2020) 10:15798. 10.1038/s41598-020-72458-w 32978428 PMC7519638

[5] Zhang HJ Chen H. A 4-year follow-up study on suicidal ideation of college students. Chinese School Hygiene. (2021) 42:1524–6.

[6] Grasdalsmoen M Eriksen HR Lønning KJ Sivertsen B. Physical exercise, mental health problems, and suicide attempts in university students. BMC Psychiatry. (2020) 20:1–11. 10.1186/s12888-020-02583-3 32299418 PMC7164166

[7] Sa J Choe CS Cho CB Chaput JP Lee J Hwang S. Sex and racial/ethnic differences in suicidal consideration and suicide attempts among US college students, 2011–2015. Am J Health Behav. (2020) 44:214–31. 10.5993/AJHB.44.2.9 32019654

[8] Matsumoto D Yoo SH Fontaine J. Mapping expressive differences around the worldthe relationship between emotional display rules and individualism vs. collectivism. J Cross Cult Psychol. (2008) 39:55–74. 10.1177/0022022107311854

[9] Li XM Luo J Gao WB Yuan J. A study on the negative emotions, coping styles, self-esteem and interpersonal relationships of college students with left-behind experience. China Linchuan J Psychol. (2009) 17:620–2.

[10] Dyrbye LN Thomas MR Shanafelt TD. Systematic review of depression. Anxiety, and other indicators of psychological distress among US and Canadian medical students Academic medicine. J Assoc Am Med Colleges. (2006) 81:354–73. 10.1097/00001888-200604000-00009 16565188

[11] Goodwin G. The overlap between anxiety depression, and obsessive-compulsive disorder. Dialogues Clin Neurosci. (2015) 17:249–60. 10.31887/DCNS.2015.17.3/ggoodwin 26487806 PMC4610610

[12] Sun SQ Wang W Kang LJ Liu ZC. To explore the social and psychological factors related to suicide attempts in college students. Nerve Damage Funct Reconstruct. (2021) 16:506–9. 10.16780/j.cnki.sjssgncj.2020103921569663

[13] Nwaoga C Nche G Nnadi FU. The pervasiveness of ebola virus disease in Africa: implication for economy, ecology and socio-religious dynamics. IOSR J Human Soc Sci. (2014) 19:69–77. 10.9790/0837-191116977

[14] Tausczik Y Faasse K Pennebaker JW Petrie KJ. Public anxiety and information seeking following the H1N1outbreak: blogs, newspaper articles, and wikipedia visits. Health Commun. (2012) 27:179–85. 10.1080/10410236.2011.571759 21827326

[15] Liang BY. Common psychological stress reaction and psychological intervention of the people during the epidemic period of SARS. Psychol Behav Stud. (2003) 1:223–30.

[16] APA. Diagnostic and Statistical Manual of Mental disorders (DSM-5). Arlington, VA: American Psychiatric Publication (2013).

[17] Arpaci I Karataş K Baloglu M. The development and initial tests for the psychometric properties of the COVID-19 Phobia Scale (C19P-S). Pers Individ Dif. (2020) 164:110108. 10.1016/j.paid.2020.110108 32394993 PMC7211675

[18] Wang C Cheng Z Yue XG McAleer M. Risk management of COVID-19 by Universities in China. J Risk Financial Manage. (2020) 13:36. 10.3390/jrfm13020036

[19] Xiao C. A novel approach of consultation on 2019 novel coronavirus (COVID-19)-Related psychological and mental problems: structured letter therapy. Psychiatry Investig. (2020) 17:175–6. 10.30773/pi.2020.0047 32093461 PMC7047000

[20] Wardenaar KJ Lim CC Al-Hamzawi AO Alonso J Andrade LH Benjet CD. The cross-national epidemiology of specific phobia in the World Mental Health Surveys. Psychol Med. (2017) 47:1744–60. 10.1017/S0033291717000174 28222820 PMC5674525

[21] Corrocher R Tedesco F Rabusin P De Sandre G. Effect of human erythrocyte stromata on complement activation. Br J Haematol. (1975) 29:235–41. 10.1111/j.1365-2141.1975.tb01817.x 33

[22] Ausín B Muñoz M Castellanos MÁ García S. Prevalence and characterization of specific phobia disorder in people over 65 years old in a Madrid community sample (Spain) and its relationship to quality of life. Int J Environ Res Public Health. (2020) 17:1915. 10.3390/ijerph17061915 32183487 PMC7143732

[23] Keyes A Deale A Foster C Veale D. Time intensive cognitive behavioural therapy for a specific phobia of vomiting: a single case experimental design. J Behav Therapy Exp Psychiat. (2020) 66:101523. 10.1016/j.jbtep.2019.101523 31706171

[24] Von Haaren B Ottenbacher J Muenz J Neumann R Boes K Ebner-Priemer U. Does a 20-week aerobic exercise training programme increase our capabilities to buffer real-life stressors? A randomized, controlled trial using ambulatory assessment. Eur. J. Appl. Physiol. (2016) 116:383–94. 10.1007/s00421-015-3284-8 26582310

[25] Dong BL Mao LJ. The effects of exercise engagement, exercise commitment, and subjective experience on college students' exercise habits: a mixed model. J Tianjin Institute Phys Edu. (2018) 33:492–9. 10.13297/j.cnki.issn1005-0000.2018.06.006

[26] Yan J Chen S Chen AG Chen JF Chen ZS. Effects of aerobics exercise and groupmental training on psychological stress and cortisol in college girls. Sports Sci. (2013) 34:81–6. 10.13598/j.issn1004-4590.2013.06.02025866255

[27] Yin JC Ji L. Can physical exercise buffer psychological stress?—a review based on Hill's seven methodological criteria. Sports Sci. (2013) 34:24–9. 10.13598/j.issn1004-4590.2013.03.012

[28] Pang WQ. The effect of physical exercise on the physical self-esteem of female normal college students. Sports J. (2017) 24:123–7. 10.16237/j.cnki.cn44-1404/g8.2017.03.016

[29] Xu W Li Y. The effect of physical exercise on depression in female college students: The multiple mediating effects of the three dimensions of social support Chinese. J Sports Med. (2017) 36:423–8. 10.16038/j.1000-6710.2017.05.008

[30] Gong X Xie XY Xu R Luo YJ. Depression-anxiety-stress scale simplified chinese version (DASS-21) test report in chinese college students China Linchuan. J Psychol. (2010) 18:443–6. 10.16128/j.cnki.1005-3611.2010.04.020

[31] Wen Y Wu DX Lv XJ. Evaluation of the reliability and validity of the Chinese version of the depression-anxiety-stress scale. Chinese Public Health. (2012) 28:1436–8.30785537

[32] Liang DQ. The stress level of college students and its relationship with physical exercise. Chinese J. Mental Health. (1994) 8:5–6.

[33] Fang J Wen ZL Liang DM. Moderating effect analysis based on multiple regression. J Psychol Sci. (2015) 38:715–20.

[34] López-Valenciano A Suárez-Iglesias D Sanchez-Lastra MA Ayán C. Impact of COVID-19 pandemic on university students' physical activity levels: an early systematic review. Front Psychol. (2021) 11:3787. 10.3389/fpsyg.2020.624567 33519653 PMC7845570

[35] Xiao P Chen L Dong XQ. Anxiety, depression, and satisfaction with life among college students in China: nine months after initiation of the outbreak of COVID-19. Front Psychiatry. (2022) 12:10. 10.3389/fpsyt.2021.777190 35126198 PMC8808246

[36] Xiang MQ Tan XM Sun J Yang HY Zhao XP Liu L. Relationship of physical activity with anxiety and depression symptoms in chinese college students during the COVID-19 outbreak. Front Psychol. (2020) 11:582436 10.3389/fpsyg.2020.582436 33329238 PMC7714784

[37] Wathelet M Duhem S Vaiva G Baubet T Habran E Veerapa E. factors associated with mental health disorders among university students in france confined during the COVID-19 pandemic. JAMA Network Open. (2020) 3:e2025591. 10.1001/jamanetworkopen.2020.25591 33095252 PMC7584927

[38] Hamlyn-Williams CC Freeman P Parfitt G. Acute affective responses to prescribed and self-selected exercise sessions in adolescent girls: an observational study. BMC Sports Sci Med Rehabil. (2014) 6:35. 10.1186/2052-1847-6-35 25285215 PMC4182279

[39] Wang YJ Lin JY Xie T Er M Wu Q Lei Q. How exercise affects adolescent depression: a review and prospect. Psychol Sci. (2021) 44:1208–15. 10.16719/j.cnki.1671-6981.20210525

[40] Wang SK Wang SJ Wang YJ Dan ZH. The effect of exercise on depression and its neurobiological mechanism research progress. Chinese General Med. (2022) 25:3443–51.

[41] Huang S Wang D Zhao J Chen H Ma Z Pan Y Liu X Fan F. Changes in suicidal ideation and related influential factors in college students during the COVID-19 lockdown in China. Psychiatry Res. (2022) 9:314. 10.1016/j.psychres.2022.114653 35671561 PMC9404404

[42] Dong RQ Zhou X Jiao XN Guo BS Sun LP Wang Q. Investigation and research on the psychological status of quarantined people during the outbreak of novel coronavirus pneumonia. J Rehabilitat. (2020) 30:7–10.

[43] Diker B Demirkan H. Distress, anxiety, boredom, and their relation to the interior spaces under COVID-19 lockdowns. Archnet-IJAR. (2022) 16. 10.1108/ARCH-03-2022-0088

[44] Di WH Zhang Q Hu W Yan J. A meta-analysis of anxiety symptoms and related factors among Chinese college students before and after the outbreak of COVID-19. Chin J Mental Health. (2022) 36:626–32.

[45] Yang SF. The effect of physical exercise on physical and mental health and its mechanism. J Beijing Sports Univ. (2011) 34:138–40. 10.19582/j.cnki.11-3785/g8.2011.06.038

[46] Berger BG Friedman E. Comparison of jogging, the relaxation response, and group interaction for stress reduction. J Sport Exercise Psychol. (1988) 10:431–47. 10.1123/jsep.10.4.431

[47] Wipfli BM Rethorst CD Landers D. The anxiolytic effects of exercise: a meta-analysis of randomized trials and dose–response analysis. J Sport Exercise Psychol. (2008) 30:392–410. 10.1123/jsep.30.4.392 18723899

[48] Liu CH. The effect of physical exercise on the negative emotions of college students: The mediating and moderating roles of self-efficacy and mental toughness. Sports J. (2020) 27:102–8. 10.16237/j.cnki.cn44-1404/g8.2020.05.014

[49] Yin JC Xue Y. The regulating effect of physical exercise on the negative emotions of college students under exam stress: A small sample daily tracking design. J Tianjin Inst Phys Edu. (2017) 32:443–7. 10.13297/j.cnki.issn1005-0000.2017.05.011

[50] Zhang CL Zhang SW Xiao KP. The effect of exercise intervention on college students' psychological stress: The mediating effect of health beliefs. J Chengdu Inst Phys Edu. (2016) 42:103–8. 10.15942/j.jcsu.2016.04.018

[51] Jiao JH Ji H Chen J. Influence of traditional Wuqinxi on physical fitness and mental health of female college students. Chinese School Hygiene. (2021) 42:1323–7. 10.16835/j.cnki.1000-9817.2021.09.011

[52] Paluska SA Schwenk TL. Physical activity and mental health sports. Medicine. (2000) 29:167–80. 10.2165/00007256-200029030-00003 10739267

[53] Li CJ Jia HN Zuo JN. The effect, mechanism and prospect of exercise in promoting mental health China. Sports Technol. (2015) 51:132–9. 10.16470/j.csst.2015.01.016

[54] Ren GQ Wang YD Zhou YB. Causal inference in individual health research—methods, applications and prospects. Population Econ. (2021) 12:12–25.

